# Complete genome sequence and comparative analysis of a *Vibrio vulnificus* strain isolated from a clinical patient

**DOI:** 10.3389/fmicb.2023.1240835

**Published:** 2023-10-31

**Authors:** Fei Wu, Tingting Zhang, Qimin Wu, Xue Li, Miaomiao Zhang, Xi Luo, Yiquan Zhang, Renfei Lu

**Affiliations:** ^1^Department of Clinical Laboratory, Nantong Third People’s Hospital, Affiliated Nantong Hospital 3 of Nantong University, Nantong, China; ^2^School of Medicine, Nantong University, Nantong, Jiangsu, China

**Keywords:** *V. vulnificus*, pan-genome, virulence, resistance, *pgl*

## Abstract

*Vibrio vulnificus* is an opportunistic, global pathogen that naturally inhabits sea water and is responsible for most vibriosis-related deaths. We investigated the genetic characteristics of *V. vulnificus* isolated from the clinical blood culture specimen of a patient with hepatitis B virus cirrhosis in 2018 (named as *V. vulnificus* VV2018) by whole genome sequencing (WGS). VV2018 belonged to a novel sequencing type 620 (ST620) and comprised two circular chromosomes, containing 4,389 potential coding sequences (CDSs) and 152 RNA genes. The phylogenetic tree of single nucleotide polymorphisms (SNPs) using 26 representative genomes revealed that VV2108 grouped with two other *V. vulnificus* strains isolated from humans. The pan-genome of *V. vulnificus* was constructed using 26 representative genomes to elucidate their genetic diversity, evolutionary characteristics, and virulence and antibiotic resistance profiles. The pan-genome analysis revealed that VV2018 shared a total of 3,016 core genes (≥99% presence), including 115 core virulence factors (VFs) and 5 core antibiotic resistance-related genes, and 309 soft core genes (≥95 and <99% presence) with 25 other *V. vulnificus* strains. The *varG* gene might account for the cefazolin resistance, and comparative analysis of the genetic context of *varG* revealed that two genes upstream and downstream of *varG* were conserved. The glycosylation (*pgl*) like genes were found in VV2018 compared with Pgl-related proteins in *Neisseria* that might affect the adherence of the strain in hosts. The comparative analysis of VV2018 would contribute to a better understanding of the virulence and antibiotic resistance profiles of *V. vulnificus*. Meanwhile much work remains to be done to better understand the function of *pgl*-like genes in *V. vulnificus*.

## Introduction

*Vibrio vulnificus* is a gram-negative, rod-shaped bacterium that is widely distributed throughout marine and brackish environments (Gulig et al., 2005). *V. vulnificus* is found in association with zooplankton, crabs, and various filter feeders such as oysters (Jones et al., 2014). *V. vulnificus* is also known as an opportunistic pathogen transmitted through the consumption of raw/undercooked seafood or by direct contact causing serious wound infections and sepsis (Baker-Austin and Oliver, 2020). Several underlying medical conditions have been identified as risk factors for *V. vulnificus* infection, including chronic liver disease, diabetes mellitus, kidney disease, autoimmune disease, hematological disorders and malignancy (Menon et al., 2014). *V. vulnificus* is responsible for more than 95% of seafood-related deaths in the United States (Haftel and Sharman, 2023). Several other risk factors contribute to the high pathogenicity of *V. vulnificus* in humans, such as the presence of a capsule, the availability of iron and possession of the *vcg* gene (Jones and Oliver, 2009). Recent studies indicate that global climate change, resulting in increased surface water temperatures, enables the global distribution and spread of *V. vulnificus* (Paz et al., 2007; Heng et al., 2017).

The pan-genome refers to the pool of genetic material that is present in a group of bacteria (Tettelin et al., 2005). It is made up of the core genome (genes shared by all strains) and the accessory genome (genes shared by some strains and not all) (Iranzadeh and Mulder, 2019), including soft core genes (≥95 and <99% presence), shell genes (≥15 and <95% presence) and cloud genes (≥0 and <15% presence). The boundaries of the core genome can be extrapolated from highly-conserved genes. Pan-genome analysis has provided new insights into interspecies differentiation and whole sets of genes shared among a group of bacteria (Medini et al., 2005; Lapierre and Gogarten, 2009). Meanwhile, a large range of genomic diversity is observed for pathogenic *V. vulnificus* strains (López-Pérez et al., 2019). Although multiple virulence factors (VFs) and antibiotic resistance profiles have been identified independently (Horseman and Surani, 2011), the diversity of VFs and resistance genes among *V. vulnificus* strains remains unknown. Despite the frequent occurrence of the pathogen, the number of cases reported are relatively low, indicating that not all strains of *V. vulnificus* are equally virulent (Strom and Paranjpye, 2000; Rosche et al., 2010).

In this work, we report the complete genome sequence of *V. vulnificus* isolated from the blood culture specimen of a clinical patient with hepatitis B virus cirrhosis in 2018 (named as *V. vulnificus* VV2018), in Nantong, Jiangsu Province, China (Wu et al., 2023), and show that this strain belonged to a novel sequence type (ST620). We characterized the genomic features of this strain to reveal the putative molecular mechanisms underlying its virulence and antibiotic resistance profiles. Furthermore, pan-genome analysis revealed the distribution of VFs and resistance-related genes among *V. vulnificus* strains. Comparative analysis revealed that the genetic context of *varG* was conserved with a sequence of approximately 3 kbp encoding *ompV*-*varG*-*nodD*. Meanwhile, using comparative analysis, we first identified putative *pgl*-like genes in VV2018, that might affect the adherence of the strain in hosts; however, much work still needs to be done to confirm this putative effect.

## Materials and methods

### Bacterial strain and genomic DNA extraction

VV2018 was isolated from the blood culture specimen of a clinical patient with hepatitis B virus cirrhosis in 2018, in Nantong, Jiangsu Province, China (Wu et al., 2023). The clinical blood sample was used with the approval of the Ethics Committee of Affiliated Nantong Hospital 3 of Nantong University. The strain was identified using the bioMérieux VITEK 2 compact instrument (bioMérieux, Marcy-l’Étoile, France) and average nucleotide identity (ANI) analysis. The genomic DNA of VV2018 was extracted using a TIANamp Bacteria DNA Kit (Tiangen Biotech Company Ltd., Beijing, China), according to the manufacturer’s protocol.

### Assessment of antibiotic resistances

The antibiotic resistance profiles were assessed through minimal inhibitory concentration (MIC) assays (Liu and Crosa, 2012). Briefly, a final suspension of 10 cfu/mL in broth supplemented with 2% NaCl and 1 mM CaCl_2_-H_2_O were distributed in triplicate throughout a 96-well microtiter plate. *Escherichia coli* ATCC 25922 was used as the susceptible-control reference bacterial strain for MIC assays. Cells were challenged with 0.25–1,024 g/mL antibiotics. MICs were determined by detection of cell pellet formation in the bottom of the wells of the 96-well plate by turbidometry at 600 nm using Multiskan GO (Thermo Fisher Scientific, USA). Drug susceptibility was determined according to the Clinical and Laboratory Standards Institute (CLSI) drug susceptibility test standard from 2018.

### Genomic DNA sequencing, assembly and annotation

Whole genome sequencing (WGS) and assembly were conducted at Azenta Life Sciences (Suzhou, China). Sequences of VV2018 were obtained using PacBio Sequel platform (Pacific Biosciences, Menlo Park, CA, USA) and Illumina HiSeq X Ten platform (Illumina, San Diego, CA, USA). The PacBio reads were assembled by Hifiasm v0.13-r308 and Canu v2.2 (Koren et al., 2017; Cheng et al., 2021), and then the Illumina reads were mapped onto the assembled contigs to correct the primary assembly and control assembly quality using Pilon 1.22 and Quiver (Chin et al., 2013; Walker et al., 2014). The genome completeness and contamination of all *V. vulnificus* strains we used were further evaluated by checkM with default settings (Parks et al., 2015). Prokka 1.14.6 was used to predict potential CDSs (Seemann, 2014). The functional annotation of these CDSs was performed by DIAMOND (Buchfink et al., 2021) against the non-redundant protein sequence (NR) database of the National Center for Biotechnology Information (NCBI). Kyoto Encyclopedia of Genes and Genomes (KEGG), Cluster of Orthologous Groups (COG), UniProt/Swiss-Prot, Pfam, CAZymes, virulence factors of pathogenic bacteria (VFDB) and Antibiotic Resistance Genes Database (ARDB) were also used to annotate the functions of CDSs (Liu and Pop, 2009; Levasseur et al., 2013; Kanehisa et al., 2017; Galperin et al., 2021; Mistry et al., 2021; Liu et al., 2022; UniProt Consortium, 2023). The rRNA and tRNA sequences were annotated by RNAmmer (Lagesen et al., 2007) and tRNAscan-SE (Lowe and Chan, 2016), respectively. The mobile genetic elements (MGEs) were annotated using ISfinder (Siguier et al., 2006). Genomic islands (GIs), prophages, and CRISPR-Cas systems were identified using online tools IslandViewer 4, PHAge Search Tool (PHAST), and CRISPRCasFinder software, respectively (Arndt et al., 2016; Bertelli et al., 2017; Couvin et al., 2018). Multilocus sequence typing (MLST) was performed by analyzing the housekeeping genes on the MLST website.^1^ The basic characteristics of the chromosomes were visualized by the CGView Comparison Tool (Petkau et al., 2010).

### Comparative sequence analysis

All the available *V. vulnificus* complete genome sequences (*n* = 25) were downloaded from the NCBI database with checkM values (Supplementary Table 1). The genome sequences were re-annotated with Prokka 1.14.6 and pan-genome analysis was conducted based on the output of Prokka using Roary with a BLASTP identity cutoff of 90% (Page et al., 2015). For genome similarity assessment, digital DNA-DNA hybridization (dDDH) values were computed using web tool GGDC 3.0 (formula 2, identities/HSP length) (Meier-Kolthoff et al., 2022). Whole genome ANI between pairwise *V. vulnificus* strains was calculated with Pyani software available at https://github.com/widdowquinn/pyani. The core genome of these strains was produced by Harvest software v1.1.2 (Treangen et al., 2014) using the *V. vulnificus* CMCP6 genome as a reference. Recombination events were removed from the core-genome alignment using Gubbins v2.2.0 (Croucher et al., 2015). Single nucleotide polymorphisms (SNPs) were then extracted from the recombination-free core genome alignment using the script available at https://github.com/sanger-pathogens/snp-sites. The multi-alignments were aligned with the ClustalW in MEGA 11.0 and analyzed using GeneDoc 2.7.0 (Nicholas and Nicholas, 1997; Tamura et al., 2021). The maximum likelihood (ML) phylogenetic tree of SNPs was constructed using RAxML in the GTRGAMMA model (1,000 bootstrap) (Stamatakis, 2014) and was visualized using Figtree v1.4.4.^2^ The neighbor-joining phylogenetic trees of PglC and PglD performed by MEGA 11.0. CD-HIT used to cluster the retained sequences using the genome sequence of VV2018 as the reference with identity of 80% and coverage of 90% (Li and Godzik, 2006).

## Results and discussion

### Genome characteristics of VV2018

**FIGURE 1:**
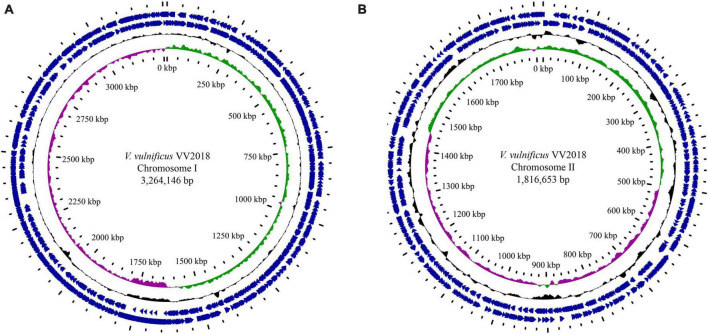
Circular genome maps of VV2018. **(A)** Chromosome I. **(B)** Chromosome I. Counting from the center toward the outside: (1) the innermost circle shows the position in kbp. (2) GC skew (G-C/G+C), with a positive GC skew toward the outside and a negative GC skew toward the inside. (3) GC content, with an average of 50%, whereby a G+C content of more than 50% is shown toward the outside, otherwise, inward. (4) Genes encoded in the leading strands (outward) or lagging strands (inward).

The complete genome of VV2018 comprised two chromosomes, Chr I and Chr II. The genome completeness of VV2018 was 100%, and the contamination was 0.05% accessed by checkM. Chr I consisted of 3,264,146 bp with a GC content of 46.60% containing 2,874 predicted CDSs, 106 tRNA genes and 31 rRNA genes. Chr II consisted of 1,816,653 bp with a GC content of 47.19% containing 1,515 predicted CDSs, 13 tRNA genes and 3 rRNA genes (Table 1 and Figure 1). MLST revealed that VV2018 belonged to a novel ST620 and was very close to ST387, with eight loci, isolated from humans in China.

**TABLE 1:** General features of VV2018.

Features	Chr I	Chr II
Length (bp)	3,264,146	1,816,653
G+C content (%)	46.60	47.19
Predicted coding sequences (CDSs)	2,874	1,515
Average length (bp)	976	1,048
Known proteins	1,787	829
Hypothetical proteins	1,087	686
Protein coding (%)	85.97	87.47
rRNA genes	31	3
tRNA genes	106	13

The distribution of VV2018 CDSs into COG functional categories is shown in Supplementary Figure 1. Except for genes with unknown functions (8.57%), most genes were related to signal transduction mechanisms, amino acid transport and metabolism, transcription, and carbohydrate transport and metabolism. The annotation of genes of VV2018 in KEGG pathway analysis showed that the most genes were involved in metabolism, including carbohydrate metabolism, amino acid metabolism, metabolism of cofactors and vitamins, and energy metabolism (Supplementary Figure 2).

### Virulence factors and resistance-related genes

A total 151 putative VFs were predicted among Chr I (106, 70.20%) and Chr II (45, 29.80%). These genes were mainly associated with motility (polar flagellar proteins), immune evasion (capsular polysaccharide and iron uptake), secretion system (type II secretion system proteins), adherence (type IV pilus, lipooligosaccharide and OmpU) and toxin (RTX toxin) (Table 2). Iron uptake from host cells plays a key role in the survival of *V. vulnificus* (Dittmann et al., 2019). RTX toxin (*rtxABCD*) and OmpU have been shown to play important roles in the infection and pathogenesis, respectively, of *V. vulnificus* (Goo et al., 2006; Liu and Crosa, 2012). The *vvhA* and *tlh* were another two toxin genes, encoding cytolysin-hemolysin and thermolabile hemolysin, which induced acute cell death and were important in the pathogenesis and dissemination of these bacteria (Wang et al., 2015; Song et al., 2016). In this case, the patient’s temperature was 40.2°C after infection, which was accompanied by chills, unbearable low back pain, and forced position. Thus, the serious infection of this case might have a strong relationship with the mixing effect of multiple VFs (Wu et al., 2023).

**TABLE 2:** The annotation of VFs of the VV2018 in VFDB databases.

Virulence factor	Gene numbers
Motility	56
Adherence	42
Immune evasion	20
Secretion system	13
Toxin	6
Regulation	1
Others	13

Six antibiotic resistance-related genes were identified in the genome of VV2018, including *dfrA3* (encoding a dihydrofolate reductase), *qnrVC1* (encoding a pentapeptide repeat protein), *catB9* (encoding a type B-5 chloramphenicol O-acetyltransferase), *tet* (Bertelli et al., 2017) (encoding a tetracycline efflux pump), *crp* (encoding a cAMP-receptor protein) and *varG* (showing resistance to penicillin, carbapenems and cephalosporins *in vitro*). CRP is a global regulator that not only regulates the expression of the multidrug efflux pump but also impacts the expression of multiple VFs (Nishino et al., 2008; Zhan et al., 2008). The antibiotic resistance pattern of VV2018 is shown in Table 3. This isolate was susceptible to most tested antibiotics, including tetracycline and chloramphenicol, with the exception of cefazolin. The fact that VV2018 showed susceptibility to tetracycline may be the result of acetylation-mediated down-regulation of *tetA* gene (Pang et al., 2020). The study also reported that all of *Vibrio cholerae* strains harboring *catB9* gene were susceptible to chloramphenicol (Lepuschitz et al., 2019). Further work needs to be done to study *catB9* in *Vibrio*. *V. vulnificus* has been reported to show complete resistance against cefazolin (Pan et al., 2013). The *varG* gene has been shown to have beta-lactamase activity against penicillin, carbapenems, and cephalosporins *in vitro* (Lin et al., 2017), which might account for the cefazolin resistance of VV2018.

**TABLE 3:** The antibiotic resistance profile of VV2018.

Antibiotics	MIC (μg/ml)	Susceptibility
Ampicillin	≤ 2	S
Cefuroxime-axetil	4	S
Cefazolin	16	R
Ceftazidime	≤ 1	S
Piperacillin	≤4	S
Imipenem	≤ 1	S
Amikacin	8	S
Meropenem	≤ 0.25	S
Gentamicin	4	S
Ciprofloxacin	≤ 0.25	S
Cefepime	≤ 1	S
Tetracycline	1	S
Chloramphenicol	1	S
Aztreonam	4	S

S, susceptible; R, resistant.

### Genomic islands, prophages and CRISPR-Cas systems

Large parts of the genome designated as genomic islands (GIs) and phages were transferred from one bacterium to another (Canchaya et al., 2003; Dobrindt et al., 2004). Twenty GIs and nine GIs were detected on Chr I and Chr II in VV2018, respectively (Supplementary Tables 2, 3). The length of GIs on Chr I ranged from 4 kbp to 163 kbp. In the GIs of Ch I, a total of 6 transposase genes were predicted, all of which were classified into the IS*4*, IS*481* and IS*5* families. Meanwhile, one integrases (*intS*) and two tyrosine recombinases (*xerC* and *xerD*) were encoded. Two genes were predicted to encode type I restriction enzyme proteins. The length of GIs on Chr II ranged from 4 kbp to 86 kbp, containing one tyrosine recombinase-encoding genes (*xerC*). Meanwhile, sulfate permease genes (*cysTWA*) which allowed the bacteria survive in selenite environment by decreasing the expression, were found on Chr II_GI2 (Tempel et al., 2022). Only one incomplete prophage sequence was predicted on Chr I with a length of 9.7 kbp encoding genes with unknown functions (Supplementary Table 4). Thus, further work needs to be done to investigate the functions of these genes.

One CRISPR locus was predicted without *Cas* genes on Chr I (Supplementary Table 5), the phenomenon that CRISPR locus without *Cas* genes was also found in other *Vibrio* strains, *Listeria monocytogenes* and *Staphylococcus*, indicating that it was unable to effectively exert adaptive immunity (Mandin et al., 2007; Zhang et al., 2019, 2021). There were four direct repeats with a length of 32 bp and three spacers. The sequences of spacers closely matched other *V. vulnificus* strains in the NCBI database.

### Comparative genome analysis of VV2018

**FIGURE 2:**
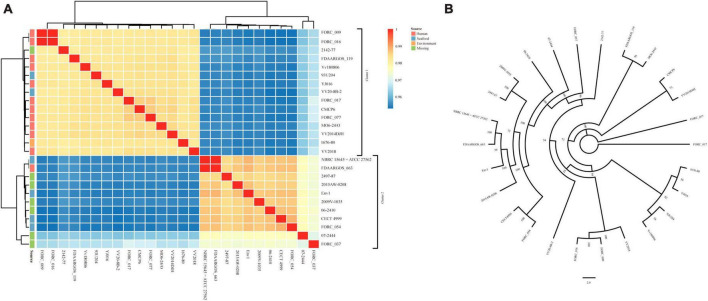
Comparative analysis between VV2018 and 25 other *V. vulnificus* strains. **(A)** Heatmap of ANI of 26 *V. vulnificus* strains. Blue color represents low identity and red color represents high identity. **(B)** An unrooted maximum-likelihood phylogeny tree of VV2018 with 25 other *V. vulnificus* strains based on core genome SNPs.

The ANI and dDDH values of 26 *V. vulnificus* strains are summarized in Supplementary Table 6. The ANI value between VV2018 and other *V. vulnificus* strains was 97.14% (range 95.41 to 98.45%) (Figure 2A). The most similar strain compared to VV2018 was *V. vulnificus* FORC_017 (98.45% identity) isolated from human in South Korea. The heatmap showed that all 26 *V. vulnificus* strains were divided into two clusters, most strains including VV2018 in cluster 1 were isolated from human, and most strains in cluster 2 were isolated from seafood or unknown places. Meanwhile, the phylogenetic tree of all 26 *V. vulnificus* strains constructed on the basis of the core SNPs showed that VV2018 was grouped with two other *V. vulnificus* strains isolated from human (*V. vulnificus* FORC_009 and *V. vulnificus* FORC_016) (Figure 2B). The dDDH values among 26 *V. vulnificus* strains were more than 60%. An ANI cut-off of around 95% did not correspond to an absolute dDDH value (70% cut-off for dDDH). A previous study reported that the value of 70% dDDH could not be used as absolute boundary, but still a gap between 60 and 70% similarity seemed to embrace clear-cut clusters of organisms, given the large extent of diversity among prokaryotes (Richter and Rosselló-Móra, 2009).

VV2018 shared a total of 3,016 core genes and 314 soft core genes with other 25 *V. vulnificus* strains according to the pan-genome analysis. A total of 138 strain-specific genes, accounting for 3.14%, were identified in VV2018. The functions of the majority of VV2018 specific genes (78.26%) were unknown, the other specific genes were involved in functional categories of replication/recombination/repair (7.97%) and cell wall/membrane/envelope biogenesis (4.36%) (Supplementary Table 7).

**FIGURE 3:**
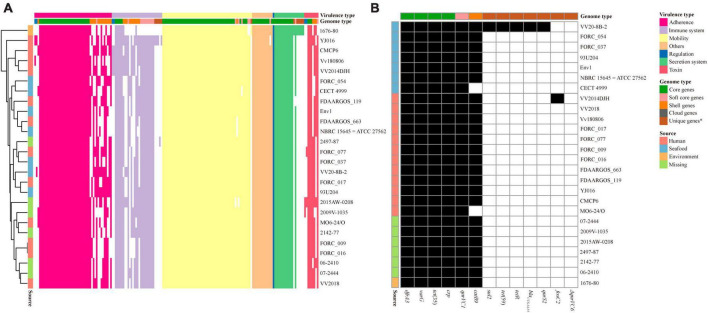
Presence/absence pattern of VFs and resistance-related genes in each *V. vulnificus* genome. **(A)** Presence/absence pattern of VFs in each *V. vulnificus* genome. **(B)** Presence/absence pattern of resistance-related genes in each *V. vulnificus* genome. *Unique genes were in only one strain included in cloud genes.

A total of 180 specific VFs were identified in 26 *V. vulnificus* strains and 115 VFs were included in core genes. The heatmap based on the presence and absence of all VFs clearly showed that the distribution of virulence genes differed between *V. vulnificus* strains (Figure 3A), and the VFs in VV2018 were similar to those in *V. vulnificus* 07-2444. Meanwhile, the differences in VFs between strains were among adherence and the immune system. In addition, some *V. vulnificus* strains isolated from humans were closely clustered with those isolated from seafood, indicating that these strains may cause foodborne infection.

Due to the misuse of antibiotics, *V. vulnificus* in seafood and aquatic environments are exhibiting resistance to multiple antibiotics (Elmahdi et al., 2016). *V. vulnificus* resistance toward common antibiotics has reached alarming levels in many countries which has serious implications for the treatment methods for bacterial infections (Heng et al., 2017). The distribution of antibiotic resistance genes in the *V. vulnificus* strains was also investigated. All 26 *V. vulnificus* strains possessed the resistance-related genes *dfrA3*, *varG*, *tet* (Bertelli et al., 2017), *qnrVC1* and *crp* (Figure 3B). The prevalence of *dfrA3*, *varG*, *tet* (Bertelli et al., 2017), *qnrVC1*, and *crp* genes in these strains suggested that these genes may increase the resistance of these strains to trimethoprim, penicillin, tetracycline, quinolone and oxacillin. The phenicol resistance gene *catB9* were also in most genomes of *V. vulnificus* strains, except *V. vulnificus* CECT 4999 and *V. vulnificus* MO6-24/O. In addition, among these strains, *V. vulnificus* VV2014DJH carried the fosfomycin resistance gene *fosC2*. Moreover, *V. vulnificus* VV20-8B-2 isolated from seafood possessed the most antibiotic resistance genes than other *V. vulnificus* strains, indicating that its antibiotic resistance may be more extensive.

### The genetic context of the *varG* gene

**FIGURE 4:**
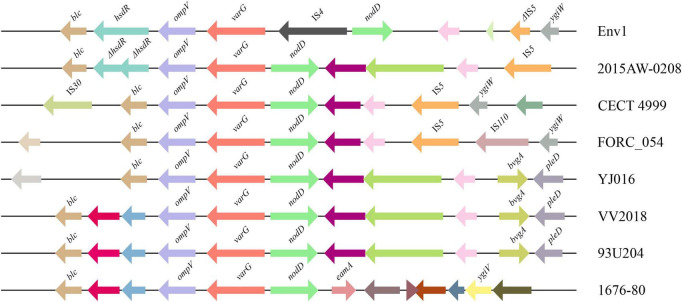
Comparative analysis of the *varG*-related regions of eight representatives from 26 sequences. The direction of genes is indicated by an arrow. Homologous genes are shown in the same colors.

The genetic context of the resistance-related genes of VV2018 was almost the same compared with other 25 *V. vulnificus* strains, except the *varG* gene. The gene *varG* might account for the cefazolin resistance of VV2018, and showed resistance to penicillin, carbapenems, and cephalosporins *in vitro* (Lin et al., 2017), however, the genetic context of *varG* was unknown in *V. vulnificus* strains. The *varG* gene was present in all 26 *V. vulnificus* strains, and the sequences containing *varG* were clustered into eight clusters with coverage of 90% and identity of 80% (Supplementary Table 8). The largest cluster was cluster 3 containing 12 sequences, and most of them (8/12, 75.0%) were isolated from humans including VV2018. Eight representative sequences were chosen for further analysis (Figure 4). The results of this gene neighborhood analysis of representative sequences revealed that a few transposase genes (IS*30*, IS*5* and IS*110*) were upstream and downstream of *varG*. The genetic context of *varG* was conserved and the genes upstream and down of *varG* were *ompV* and *nodD* that were present in 96.15% of the sequences. These observations indicated that sequence rearrangement rarely occurs in the *varG*-encoding region, and genetic commonalities of *ompV*-*varG*-*nodD* across sources strongly suggested the structure of *ompV*-*varG*-*nodD* was conserved in *V. vulnificus* strains.

### Molecular characterization and comparative analysis of *pgl*-like genes

**FIGURE 5:**
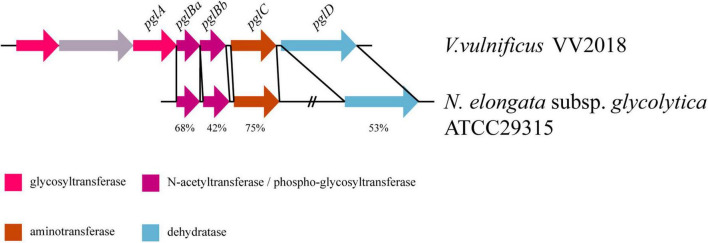
Comparative *pgl* gene content and synteny between VV2018 and *N. elongata* subsp. *glycolytica* strain ATCC29315. The direction of genes is indicated by an arrow. Homologous genes are shown in the same colors.

Two (LNNJENCE_00235 and LNNJENCE_00237) of six genes on Chr I_GI4 were glycosyl transferases, belonging to the GT4 family (Supplementary Table 9). LNNJENCE_00237 was compared with PglA in *Neisseria* including *N. elongata* subsp. *glycolytica*, with 37% identity. Meanwhile, four genes downstream of LNNJENCE_00237 also shared a high identity compared with Pgl-related proteins in *Neisseria* such as *N. elongata* subsp. *glycolytica* ATCC29315 (Table 4 and Figure 5). Broad-spectrum O-linked protein glycosylation (*pgl*) systems have been defined in *Neisseria*, such as *N. gonorrhoeae*, *N. meningitidis*, and *N. elongata* subspecies *glycolytica* (Ku et al., 2009; Vik et al., 2009; Naess et al., 2023). The *pglA* and *pglBCD* may be involved in pilin glycosylation (Power et al., 2003).

**TABLE 4:** The result of BLASTP of Pgl in VV2018 against Pgl in *Neisseria*.

Gene id	Protein name	Coverage (%)	Identity (%)	Strains of *Neisseria*
LNNJENCE_00237	PglA	97%	37%	*N. meningitidis* G2136
LNNJENCE_00238	PglBa	97%	68%	*N. elongata* subsp. *glycolytica* ATCC29315
LNNJENCE_00239	PglBb	88%	42%	*N. elongata* subsp. *glycolytica* ATCC29315
LNNJENCE_00240	PglC	99%	75%	*N. elongata* subsp. *glycolytica* ATCC29315
LNNJENCE_00241	PglD	97%	53%	*N. elongata* subsp. *glycolytica* ATCC29315

**FIGURE 6:**
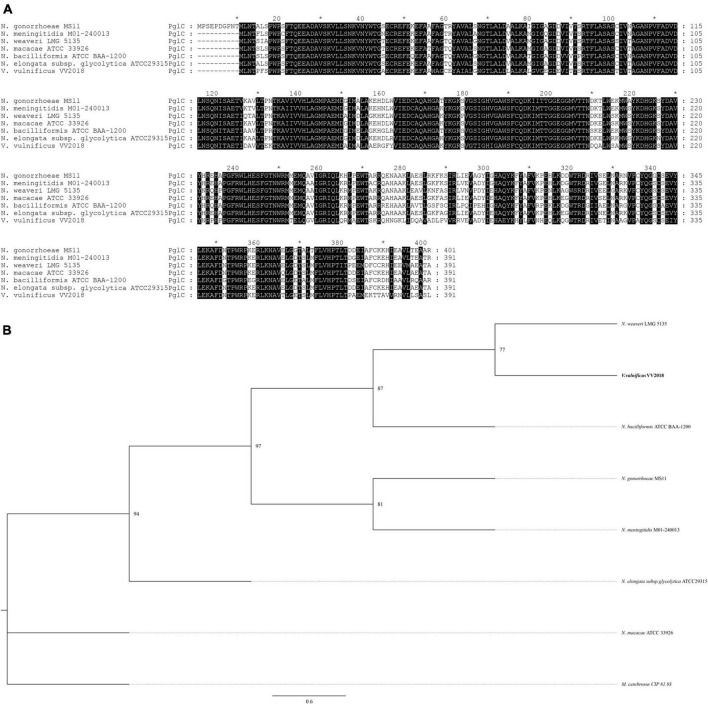
Comprehensive comparisons of PglC by sequence similarity and phylogenetic analysis. **(A)** Multisequence alignment of PglC with amino acids. Multisequence alignment was conducted using ClustalW. **(B)** A neighbor-joining phylogenetic tree of PglC was estimated by MEGA, and the sequence of *Morococcus cerebrosus* (*M. cerebrosus*) CIP 81.93 was used as outgroup. *The markers of length such as 10, 30, that are provided by software.

**FIGURE 7:**
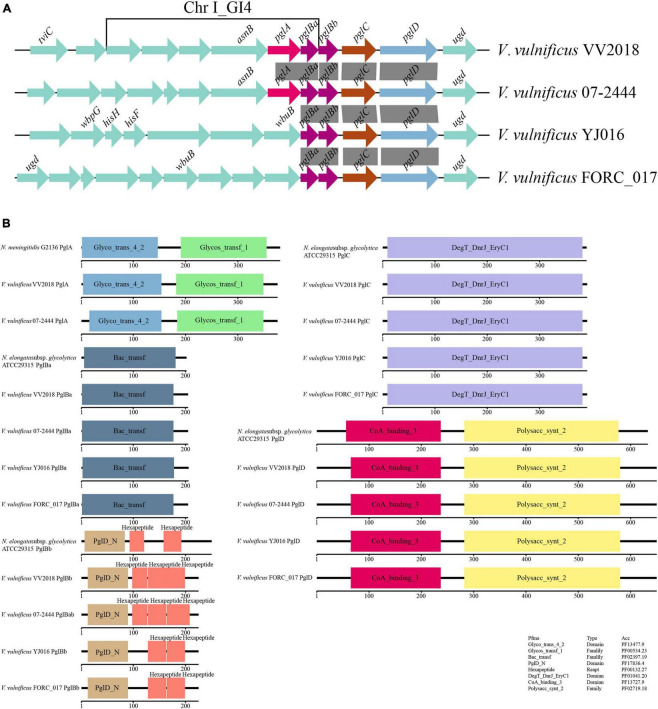
Comparative genomic analysis of *pgl*-like genes. **(A)** Comparative genomic analysis of the genetic context of *pgl* genes in VV2018 with the sequences carrying their homologous genes in three other *V. vulnificus* strains. The direction of genes is indicated by an arrow. Homologous *pgl* genes are shown in the same colors, syntenic regions between the sequences are displayed as gray blocks. All other genes are shown in green. **(B)** Schematic diagram depicting the total domain structure of PglA, PglBa, PglBb, PglC and PglD. Each scaled box denotes a functional module labeled with the term short name.

We selected six sequences of *Neisseria* for further analysis and the result of the multiple sequence alignment showed that the sequences of PglC and PglD were highly conserved (Figure 6A and Supplementary Figure 3A). The phylogenetic tree of PglC and PglD showed that the PglC of VV2018 was closest to the protein of *N. weaveri* LMG 5135, and PglD of VV2018 was closest to *N. bacilliformis* ATCC BAA-1200 and *N. elongata* subsp. *glycolytica* ATCC29315 (Figure 6B and Supplementary Figure 3B). Meanwhile, comparative genomic analysis revealed that the sequence of VV2018 containing *pgl*-like genes was similar to the sequences of *V. vulnificus* 07-2444, *V. vulnificus* YJ016 and *V. vulnificus* FORC_017 (Figure 7A). The result showed that the *pglA* gene was only found in *V. vulnificus* 07-2444 with coverage of 76% and identity of 85%. The other genes *pglBa*, *pglBb*, *pglC*, and *pglD* also had high homology with identity ranging from 90 to 100%. In addition, in the genome of *V. parahaemolyticus*, the *pglB2* gene is associated with a *pglC* and *pglD* homolog, suggesting that a complete glycosylation system might also be present (Chamot-Rooke et al., 2007). In *V. cholerae*, O-glycosylation via PglL_Vc_ and possibly RbmD could represent a fine-tuned feedback mechanism controlling release of type II secretion system (T2SS) effectors by modulation of secretion efficacy (Vorkapic et al., 2019). Therefore, *pgl*-like genes (*pglABCD*) may play important roles in *Vibrio* species.

The total domain structures of PglA, PglBa, PglBb, PglC and PglD were also analyzed and compared with protein sequences in *Neisseria*. The domains of PglA, PglBa, PglC and PglD were similar, except for PglBb (Figure 7B). The PglD_N domain was found in the N-terminus of all PglBb sequences, besides the number and location of hexapeptides. There were three hexapeptides in PglBb of VV2018 and *V. vulnificus* 07-2444. The *pgl*Bb of *V. vulnificus* YJ016 and *V. vulnificus* FORC_017 had two hexapeptides in the second and third position. The *pgl*Bb of *N. elongata* subsp. *glycolytica* strain ATCC29315 had two hexapeptides in the first and third position. A number of different transferase protein families contain hexapeptide repeats, such as galactoside acetyltransferase-like proteins (Wang et al., 2002). It has been shown that most hexapeptide acyltransferases form catalytic trimers with three symmetrical active sites (Bergfeld et al., 2007). This is the first study to report that *pglA* and *pglBCD* genes were found in *V. vulnificus* strains. The *pgl*-like genes may affect the adherence of the strain, however, much more work is need to prove this hypothesis.

## Conclusion

In this study, we investigated the genomic features of VV2018 with novel strain ST620 that was isolated from the blood culture specimen of a clinical patient with hepatitis B virus cirrhosis in China. Multiple VFs and resistance genes were identified in the genome sequence of VV2018. Pan-genome analysis of 26 *V. vulnificus* strains revealed their pan-genome characteristics, evolutionary relationships, and virulence and antibiotic resistance profiles. This study provides a snapshot of the genomic diversity and evolution of different strains that contribute to the pathogenic diversity of 26 *V. vulnificus* strains. We also found that the resistance gene *varG* was present in all 26 *V. vulnificus* strains and the genes upstream and downstream were conserved. In addition, it’s the first to report the presence of *pgl*-like genes in *V. vulnificus* based on amino acid sequence homologies with genes in *Neisseria*. The *pgl*-like genes may affect the adherence of the strain in hosts, and much work still needs to be done to confirm this theory.

## Data availability statement

The datasets presented in this study can be found in online repositories. The names of the repository/repositories and accession number(s) can be found in this article/Supplementary material.

## Ethics statement

The studies involving humans were approved by the Ethics Committee of Affiliated Nantong Hospital 3 of Nantong University. The studies were conducted in accordance with the local legislation and institutional requirements. The participants provided their written informed consent to participate in this study.

## Author contributions

TZ, QW, XuL, and XiL collected the strains and performed the experiments. MZ and TZ analyzed the experimental results. FW performed the bioinformatic analysis. FW, YZ, and RL co-led the writing of the manuscript. YZ and RL designed the work. All authors read and approved the final manuscript.

## Footnotes

1
http://pubmlst.org/vvulnificus/

2
http://tree.bio.ed.ac.uk/software/figtree/
