# Supplementary material for: Complete genome sequence and comparative analysis of a Vibrio vulnificus strain isolated from a clinical patient
Source: Front Microbiol. 2023 Oct 31;14:1240835. doi: 10.3389/fmicb.2023.1240835 (PMC10644004; doi:10.3389/fmicb.2023.1240835)
Supplement: Supplementary file 1 [file Data_Sheet_1.docx]

Table S1. Information concerning *V. vulnificus* strains from the NCBI database.

| Strain | Source | Country | Accession number | Completeness/ contamination |
| --- | --- | --- | --- | --- |
| *V. vulnificus* 06-2410 | Missing | Missing | CP046832.1/CP046833.1 | 100%/0.43% |
| *V. vulnificus* 07-2444 | Missing | America | CP046834.1/CP046835.1 | 100%/0.32% |
| *V. vulnificus* NBRC 15645 = ATCC 27562 | Seafood | America | CP012881.1/ CP012882.1 | 100%/0.3% |
| *V. vulnificus* 1676-80 | Environment | America | CP051117.1/CP051118.1 | 100%/0.58% |
| *V. vulnificus* 2009V-1035 | Missing | America | CP035783.1/CP035784.1 | 99.57%/0% |
| *V. vulnificus* 2015AW-0208 | Missing | America | CP046757.1/CP046758.1 | 97.72%/0.67% |
| *V. vulnificus* 2142-77 | Missing | America | CP035731.1/CP035732.1 | 99.93%/0% |
| *V. vulnificus* 2497-87 | Missing | America | CP060047.1/CP060048.1 | 100%/0.13% |
| *V. vulnificus* 93U204 | Seafood | China | CP009261.1/CP009262.1/  CP009263.1 | 100%/0.39% |
| *V. vulnificus* CMCP6 | Human | South Korea | AE016795.3/AE016796.2 | 100%/0.17% |
| *V. vulnificus* CECT 4999 | Seafood | Spain | CP014636.1/CP014637.1/  CP014638.1 | 100%/0.1% |
| *V. vulnificus* Env1 | Seafood | America | CP017635.1/CP017636.1 | 99.98%0.57% |
| *V. vulnificus* FDAARGOS_119 | Human | America | CP014048.2/CP014049.2 | 100%/0.03% |
| *V. vulnificus* FDAARGOS_663 | Human | America | CP044068.1/CP044069.1 | 100%/0.3% |
| *V. vulnificus* FORC_009 | Human | South Korea | CP009984.1/ CP009985.1 | 100%/0.54% |
| *V. vulnificus* FORC_016 | Human | South Korea | CP011775.1/CP011776.1 | 100%0.54% |
| *V. vulnificus* FORC_017 | Human | South Korea | CP012739.1/CP012740.1/  CP012741.1 | 100%/0.33% |
| *V. vulnificus* FORC_037 | Seafood | South Korea | CP016321.1/CP016322.1/  CP016323.1 | 100%/0.15% |
| *V. vulnificus* FORC_054 | Seafood | South Korea | CP019121.1/CP019122.1/  CP019123.1 | 100%/0.37% |
| *V. vulnificus* FORC_077 | Human | South Korea | CP027030.1/CP027031.1 | 100%/0.33% |
| *V. vulnificus* MO6-24/O | Human | South Korea | CP002469.1/CP002470.1 | 100%/0% |
| *V. vulnificus* Vv180806 | Human | China | CP044206.1/CP044207.1/  CP044208.1 | 100%/0.14% |
| *V. vulnificus* VV20-8B-2 | Seafood | Japan | AP026552.1/AP026553.1/  AP026554.1 | 100%/0.86% |
| *V. vulnificus* VV2014DJH | Human | China | CP019320.1/CP019321.1 | 99.73%/0% |
| *V. vulnificus* YJ016 | Human | China | BA000037.2/BA000038.2/  AP005352.1 | 100%/0.14% |


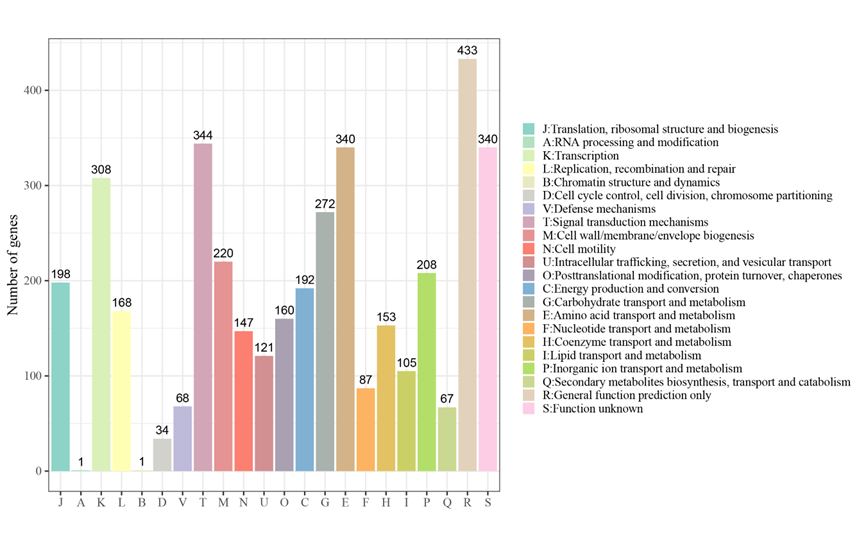


Figure S1. COG functional annotation of CDSs in the whole genome of VV2018.**
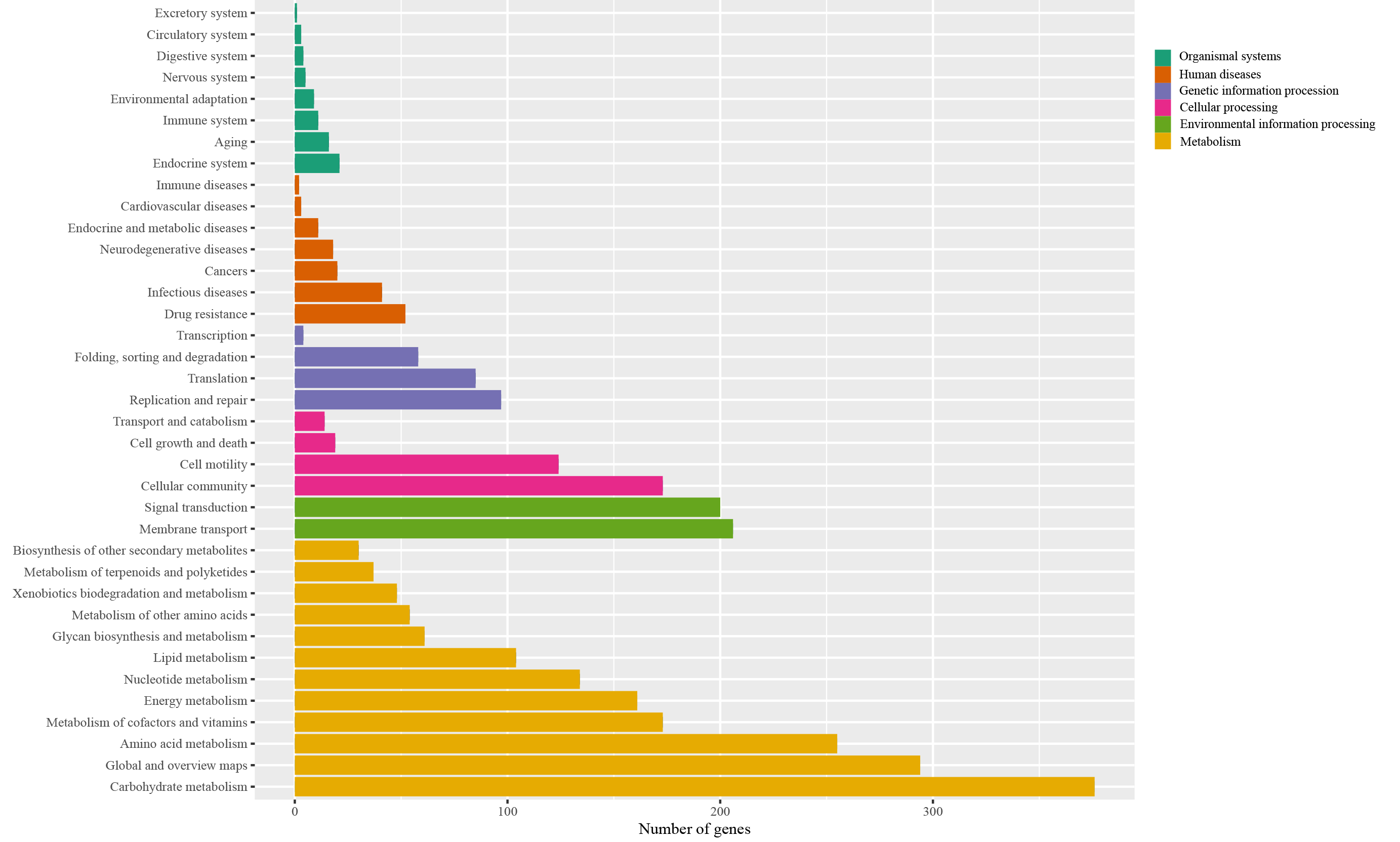
**

Figure S2. KEGG pathway annotation of CDSs in the whole genome of VV2018.

Table S7. The function of 138 strain-specific genes of VV2018.

| Function | Number |
| --- | --- |
| Amino acid transport and metabolism | 1 |
| Cell wall/membrane/envelope biogenesis | 6 |
| General function prediction only | 4 |
| Intracellular trafficking, secretion, and vesicular transport | 1 |
| Cell motility | 1 |
| Energy production and conversion | 1 |
| Nucleotide transport and metabolism | 1 |
| Lipid transport and metabolism | 1 |
| Posttranslational modification, protein turnover, chaperones | 3 |
| Replication, recombination and repair | 11 |
| Function unknown | 108 |

Table S8. Twenty-six sequences containing the *varG* gene clustered by CD-HIT with 80% identity and 90% coverage.

| Strain | Cluster |
| --- | --- |
| *V. vulnificus* 06-2410 | Cluster2 |
| *V. vulnificus* 07-2444 | Cluster2 |
| *V. vulnificus* NBRC 15645 = ATCC 27562 | Cluster7 |
| *V. vulnificus* 1676-80 | Cluster8 |
| *V. vulnificus* 2009V-1035 | Cluster3 |
| *V. vulnificus* 2015AW-0208 | Cluster2 |
| *V. vulnificus* 2142-77 | Cluster3 |
| *V. vulnificus* 2497-87 | Cluster2 |
| *V. vulnificus* 93U204 | Cluster5 |
| *V. vulnificus* CMCP6 | Cluster3 |
| *V. vulnificus* CECT 4999 | Cluster6 |
| *V. vulnificus* Env1 | Cluster1 |
| *V. vulnificus* FDAARGOS_119 | Cluster2 |
| *V. vulnificus* FDAARGOS_663 | Cluster7 |
| *V. vulnificus* FORC_009 | Cluster3 |
| *V. vulnificus* FORC_016 | Cluster3 |
| *V. vulnificus* FORC_017 | Cluster3 |
| *V. vulnificus* FORC_037 | Cluster3 |
| *V. vulnificus* FORC_054 | Cluster7 |
| *V. vulnificus* FORC_077 | Cluster3 |
| *V. vulnificus* MO6-24/O | Cluster4 |
| *V. vulnificus* Vv180806 | Cluster3 |
| *V. vulnificus* VV20-8B-2 | Cluster3 |
| *V. vulnificus* VV2018 | Cluster3 |
| *V. vulnificus* VV2014DJH | Cluster3 |
| *V. vulnificus* YJ016 | Cluster4 |

Table S9. The function of eleven genes besides *pgl*-like genes.

| Gene id | Island id | CAZyme | Function | Gene name |
| --- | --- | --- | --- | --- |
| LNNJENCE_00232 |  |  | NAD-dependent epimerase/dehydratase family protein |  |
| LNNJENCE_00233 | Chr Ⅰ_GI4 |  | Hypothetical proteins |  |
| LNNJENCE_00234 | Chr Ⅰ_GI4 |  | Hypothetical proteins |  |
| LNNJENCE_00235 | Chr Ⅰ_GI4 | GT4 | Glycosyltransferase, 2.4.1.- |  |
| LNNJENCE_00236 | Chr Ⅰ_GI4 |  | Asparagine synthase | *asnB* |
| LNNJENCE_00237 | Chr Ⅰ_GI4 | GT4 | N, N'-diacetylbacillosaminyl-diphospho-undecaprenol alpha-1,3-N-acetylgalactosaminyltransferase, 2.4.1.290 | *pglA* |
| LNNJENCE_00238 | ChrⅠ_GI4 |  | Pilin glycosylation protein PglB | *pglBa* |
| LNNJENCE_00239 |  |  | Pilin glycosylation protein PglB | *PglB*b |
| LNNJENCE_00240 |  |  | Pilin glycosylation protein, 2.6.1.- | *pglC* |
| LNNJENCE_00241 |  |  | Pilin glycosylation protein PglD | *pglD* |
| LNNJENCE_00242 |  |  | UDP-glucose 6-dehydrogenase, 1.1.1.22 | *ugd* |


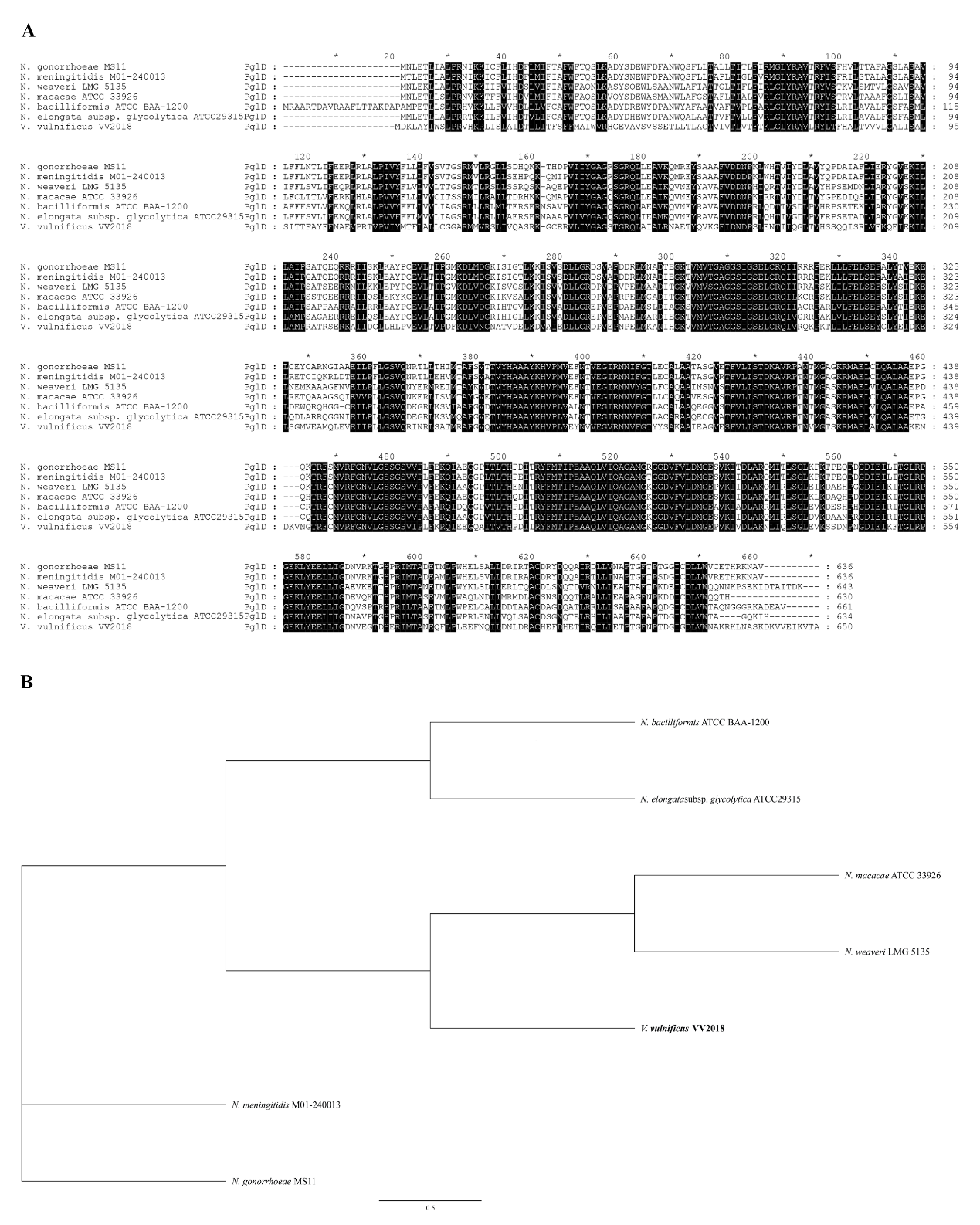


Figure S3. Comprehensive comparisons of PglD at the sequence similarity, and phylogenetic analysis. (A) Multisequence alignment of PglD with amino acids. Multisequence alignment was conducted by ClustalW. (B) A neighbor-joining phylogenetic tree of PglD was estimated by MEGA, and the sequence of M. cerebrosus CIP 81.93 was used as outgroup.
